# Cyclobutane-Containing Alkaloids: Origin, Synthesis, and Biological Activities

**DOI:** 10.2174/1874104500802010026

**Published:** 2008-04-15

**Authors:** Anastasia Sergeiko, Vladimir V Poroikov, Lumir O Hanuš, Valery M Dembitsky

**Affiliations:** 1 Institute of Biomedical Chemistry, Russian Academy of the Medical Sciences, Moscow 119121, Russia; 2 Department of Medicinal Chemistry and Natural Products, School of Pharmacy, P.O. Box 12065, The Hebrew University of Jerusalem, Jerusalem 91120, Israel

**Keywords:** Alkaloids, cyclobutane-containing, anticancer, antibacterial, synthesis, activities, terrestrial, marine.

## Abstract

Present review describes research on novel natural cyclobutane-containing alkaloids isolated from terrestrial and marine species. More than 60 biological active compounds have been confirmed to have antimicrobial, antibacterial, antitumor, and other activities. The structures, synthesis, origins, and biological activities of a selection of cyclobutane-containing alkaloids are reviewed. With the computer program PASS some additional biological activities are also predicted, which point toward new possible applications of these compounds. This review emphasizes the role of cyclobutane-containing alkaloids as an important source of leads for drug discovery.

## INTRODUCTION

Organic  compounds containing four-membered ring(s) represent unusual group of metabolites including natural products and/or drugs. The cyclobutane unit is found as a basic structural element in a wide range of naturally occurring compounds in bacteria, fungi, plants, and marine invertebrates. It is also generated transiently in primary and secondary metabolisms [1]. Many biological activities are showed and may serve as potential drug leads or provide new ideas for the study of enzyme mechanisms, and/or organic synthesis [2]. Some cyclobutane compounds such as amino acids, peptides and nucleosides showed protective properties against UV radiation [3]. In the skin many molecules may absorb UV radiation upon exposure. In particular, cellular DNA strongly absorbs shorter wavelength solar UV radiation, resulting in various types of DNA damage. Among the DNA photoproducts produced the cyclobutane pyrimidine dimers are predominant [4].

Although cyclobutanes have been known for more than a century, their use as synthetic intermediates has only flourished in the last forty years. The structures and syntheses of cyclobutanoid fatty acids, amino acids, mono-, sesqui-, di-, and triterpenes, steroids, and other compounds have recently been reported [2,5,6].

In the present review, we will focus on origin, structures, and biological activities of natural cyclobutane-containing alkaloids and selected related compounds. Their structure and biological activities, modes of action, and future prospects are discussed.

This paper is a short survey of cyclobutane-containing alkaloids that are deemed as naturally occurring. Also, this is the first article to review natural alkaloids comprising a cyclobutane unit.

## TERRESTRIAL CYCLOBUTANE-CONTAINING ALKALOIDS

GC-MS was used to analyze volatiles in the headspace of 'Fortress' onion bulbs inoculated with *Fusarium oxysporum*, *Botrytis allii, Erwinia carotovora* subsp. *carotovora*, *Aspergillus niger*, or *Penicillium aurantiogriseum*. Among 130 volatile metabolites of 'Fortress' onion bulbs were found a simple ethylcyclobutane and 2-azabicyclo[3.2.0]hept-6-ene (**1**) [7]. 2,4-Methanoproline (2-carboxy-2,4-methanopyrrolidine) (**2**) and 2,4-methanoglutamic acid (1-amino-1,3-dicarboxycyclobutane) were isolated from seeds of *Ateleia herbert smithii* (Leguminosae) [8].

The non-protein amino acids of the legume genus *Bocoa* (Papilionoideae; Swartzieae) were surveyed by LC-MS and GC-MS using extracts of herbarium leaf fragments. 2,4-Methanoproline (**2**) have also been detected in *Ateleia herbert smithii,* *Bocoa alterna*, B*. decipiens, B. limae, B. mollis*, and *B. racemulosa* [9]. Compound (**2**) was formerly suggested to have insect repellent/antifeedant activity [10].

**Scheme 1:**
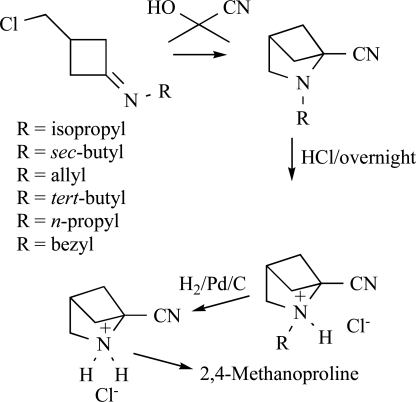


Synthesis of 2,4-methanoproline and derivatives have been reported by several authors. Most of the syntheses of 2,4-methanoproline are accomplished by an intramolecular light-induced [2+2] cycloaddition of an appropriate diene [11,12]. Only Gaoni used an intramolecular cyclization strategy for his approach [13]. Also analogues containing the 2-azabicyclo[2.1.1]hexane skeleton are often synthesized by the light-induced [2+2] cyclization [14-17]. The rearrangement of an appropriate bromohydrine to synthesize 5-hydroxy-2-azabicyclo[2.1.1]-hexanes has also been described [18,19]. A two-step synthetic approach toward 3-(chloromethyl)cyclobutanone was described and used in the synthesis of 2,4-methanoproline analogues [20]. The key step consists of a reversible addition of hydrogen cyanide onto the imines (Scheme **1**).

Seven Lycopodium alkaloids, lannotinidines A-G, have been isolated from the club moss *Lycopodium annotinum* and *L. annotinum* var. *acrifolium*. Two lannotinidines F (**3**) and E (**4**) contain cyclobutane ring elevated NGF mRNA expression. Effects of lannotinidines A–F on neurotrophic factor biosynthesis in 1321N1 human astrocytoma cells were examined by determining NGF mRNA expression. 1321N1 cells were incubated with 30 μg/mL each of lannotinidines A–F for 6 h, and the mRNA expressions of NGF in 1321N1 cells were examined by a semiquantitative RT-PCR method. The mRNA expressions for NGF were enhanced by lannotinidines B–E, among which (**3**) was the most potent [21].


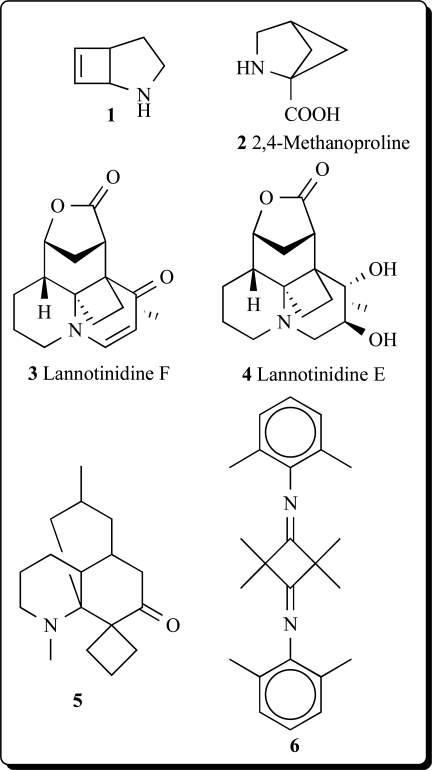


Bioactive hexahydro-1',10'-dimethyl-spiro[cyclo-butane-1,8'(7'H)-[1H-5,8a]propane-quinolin]-7'-one (**5**) was isolated from extract of *Lycopodium* species [22]. Unusual tetramethyl-N,N-*bis*(2,6-dimethyl-phenyl)-cyclobutane-1,3-diimine (**6**), triacontanol, and tricin, reported from *Arundo donax*, revieled significant antifeedant activity against the boll weevil, *Anthonomus grand *[23].

*Sarcomelicope megistophylla* (Rutaceae) is a small to medium sized tree, 8–12 m high, easily recognized by its pubescent leaves, exceptionally large for the genus (up to 35 cm long). This species is endemic to the region of Néaoua, New Caledonia [24]. A new quinolone, cyclomegistine (**7**), was isolated from the bark of *Sarcomelicope megistophylla*. This alkaloid possesses the cyclobuta[b]quinoline ring system that has not been previously described either from natural or from synthetic origin. Biogenetically, cyclomegistine could arise from the oxidative aromatic ring cleavage of an acridone precursor, followed by photo-isomerization of the resulting butadiene into the isomeric cyclobutane [25].


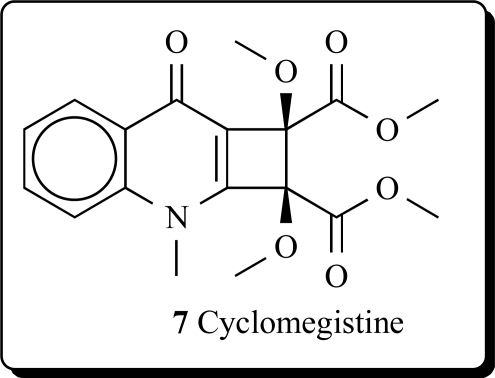


A tropane alkaloid with a 2-methyl-4-phenyl-cyclobutane 1,2,3-tricarboxylic acid ester as the central structure was isolated from the aerial parts of *Schizanthus grahamii* and named grahamine (**8**) [26].


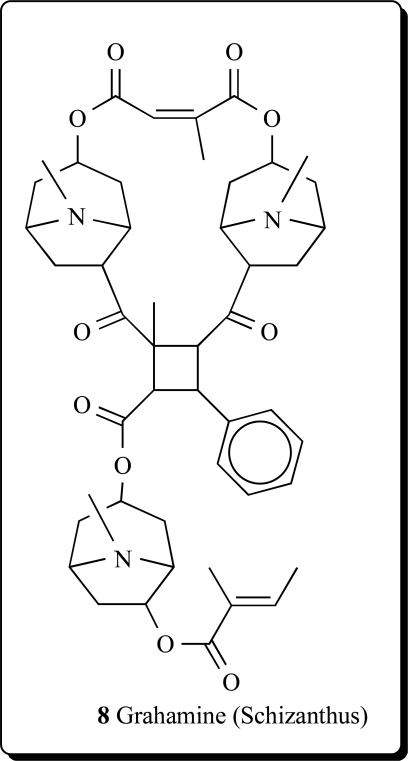


Two new dimeric tropane alkaloids, mooniines A (**9**) and B (**10**) were identified from the leaves of *Erythroxylum moonii* [27].

*Adenocarpus complicatus* subspecies *aureus *(Papilionacea) yielded alkaloids: (+)-santiaguine (0.14%, **11**), racemic santiaguine (0.01%), (+)-adenocarpine (0.23%), and 
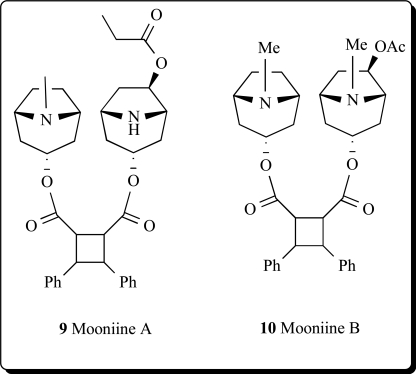
 isoorensin (0.18% of dried plant) [28]. Santiaguine has also been isolated from *Adenocarpus mannii,* *Adenocarpus foliosus*, *Adenocarpus intermedius* and *A. parvifolius* [29-40].


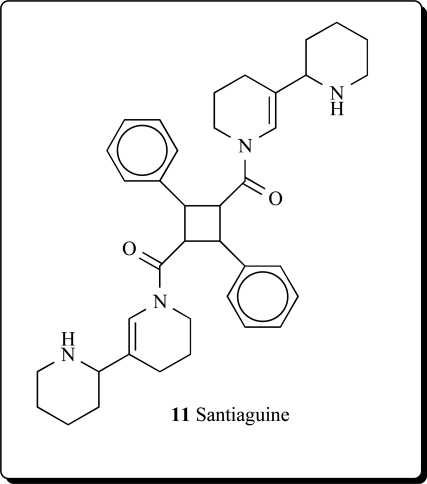


Structures of monoterpene alkaloid derivatives and spermine related compounds (**12**,**13**) isolated from *Incarvillea sinensis* were detected by physico-chemical methods. Among them, incarvilateine (**13**) showed strongest analgesic and sedative activities [41,42]. Methoxyincarvillateine (**14**), and the *N*-oxides (**15**) with similarly structure have been obtained from extract of the *Incarvillea sinensis* [43].

**Scheme 2:**
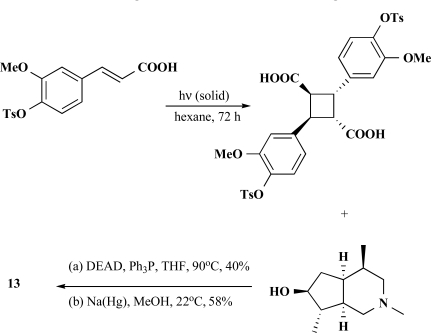


The [2+2] photodimerization of *trans*-cinnamic acids in the crystalline state gave R-truxillic acid. Condensation of the R-truxillic acid with 2 equiv of the above-described (+)-6-*epi*-incarvilline under Mitsunobu conditions produced tosyl-protected incarvillateine (Scheme **2**). Deprotection of the tosyl groups using sodium amalgam in methanol provided (-)-incarvillateine (**13**) as colorless crystals. The optical rotation and spectroscopic properties (^1^H and ^13^C NMR) of synthetic (**13**) were in agreement with those reported for natural incarvillateine [44].

A re-examination of the leaf and seed alkaloids of *Lupinus cosentinii*, confirmed in both parts the presence of epilupinine, epilupinine N-oxide, and multiflorine. Both organs of the plant contained a new tricyclic alkaloid (**16**) [45].


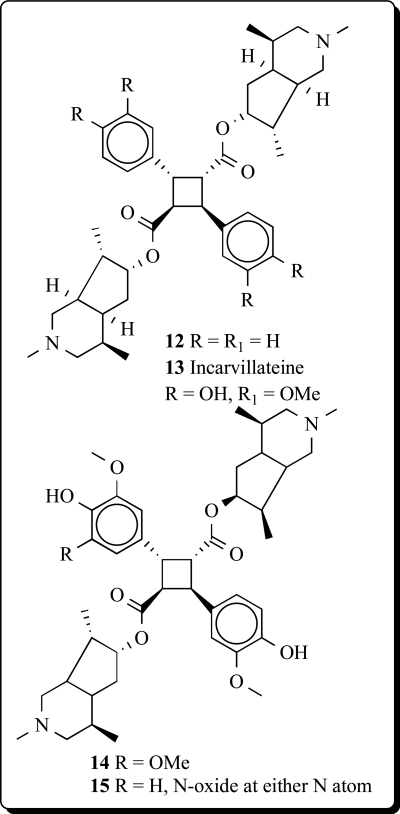


Thesine (**17**), isolated from *Thesium minkwizianum* and its water-soluble sulfate, showed extremely toxic properties; the initial oral dosage should be about 10-20 mg [46-48].

The genus *Piper *(Piperaceae) includes more than 1000 species making it one of the largest genera of basal angiosperms [49]. *Piper *species are distributed pantropically and are in the form of shrubs, herbs, and lianas common in the understory of lowland wet forests. The greatest diversity of *Piper *species occurs in the American tropics (700 spp.), followed by Southern Asia (300 spp.), where the economically important species *Piper nigrum *(Black pepper) and *P. betle* 
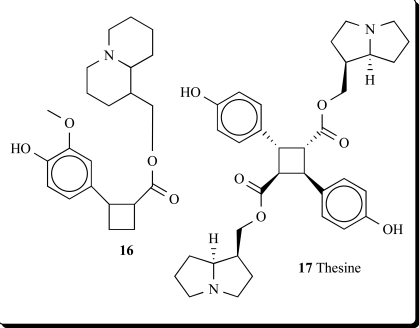
 (betel leaf) originated. Patterns of distribution of *Piper *species vary from being locally endemic to widespread. Several cyclobutane-containing alkaloids have been isolated and identified from the genus *Piper* [50]. Fifteen novel dimeric amide alkaloids possessing a cyclohexene ring, nigramides A-O, as well as four novel dimeric amide alkaloids possessing a cyclobutane ring, nigramides P-S (**18-21**), have been isolated from the roots of *Piper nigrum*. The biosynthetic hypothesis of nigramides A-O was proposed by an intermolecular Diels-Alder reaction from the corresponding monomeric amides [51].


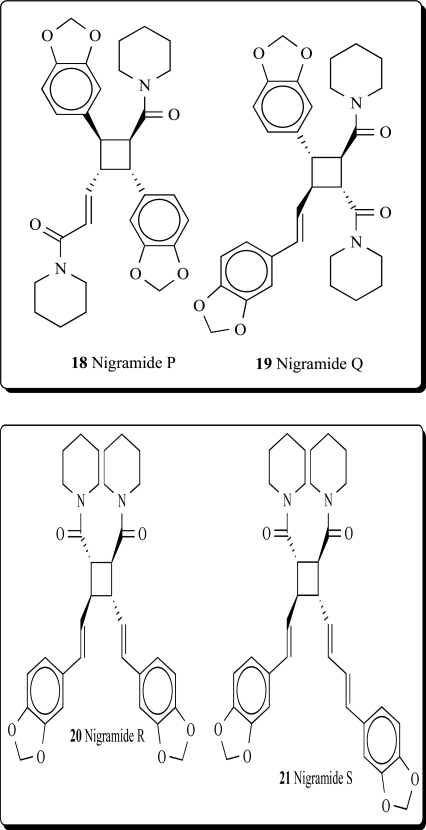


Two other new alkaloids possessing a cyclobutane ring, named pipercyclobutanamides A (**22**) and B (**23**), have been isolated from the fruits of *Piper nigrum* [52]. Isolated compounds were potent mechanism-based inhibitors of cytochrome P 450 2D6 (CYP2D6) [53,54].


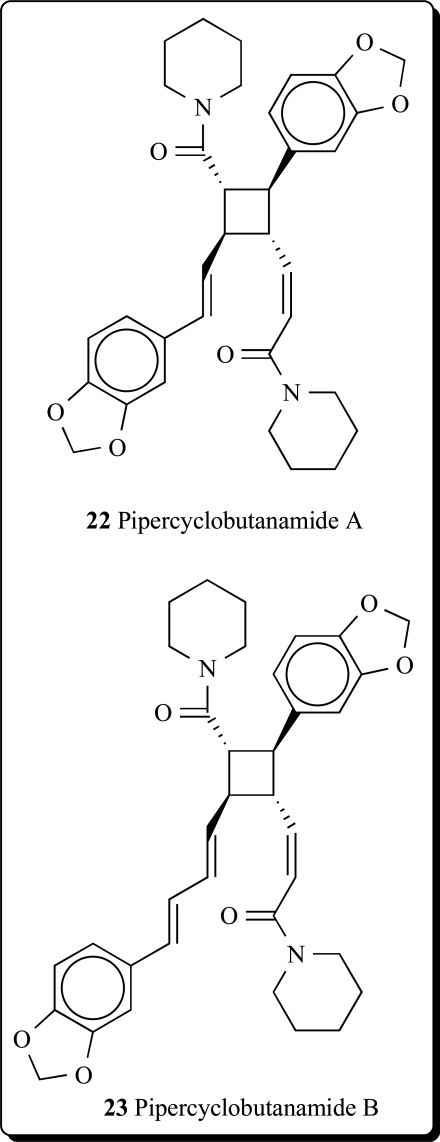


Two new cyclobutanoid amides, piplartine-dimer A (**24**), piperarborenine A (**25**) and B (**26**), and piperarboresine (**27**) were isolated from the stem of *Piper arborescens* [55]. Piplartine dimer A (**24**) has also been obtained from extracts of three piper species: *P. Aborescens, P. rugosum, P. tuberculatum* [56-58].

Three new cyclobutanoid amides with trans-trans configurations, piperarborenine C (**28**), piperarborenine D (**29**) and piperarborenine E (**30**), and a new furanoid lignan, (+)-arborone, together with twelve known compounds, were isolated from the stems of *Piper arborescens*. Piperarborenine C, (+)-diayangambin, piplartine, piperolactam B, piperolactam C, aristolactam BIII, goniothalactam, and methyl *trans*-3,4,5-trimethoxycinnamate possessed anti-platelet aggregation activity *in vitro*. Among them, piplartine showed the most potent anti-platelet aggregation activity induced by collagen and showed an IC_50_ value of 21.5 μM. Piperarborenines C-E, piperarborenine, aristololactam BIII and goniothalactam showed significant cytotoxic activity (IC_50_ values < 4 μg/mL) against P-388, HT-29 and A549 cell lines *in vitro* [59].


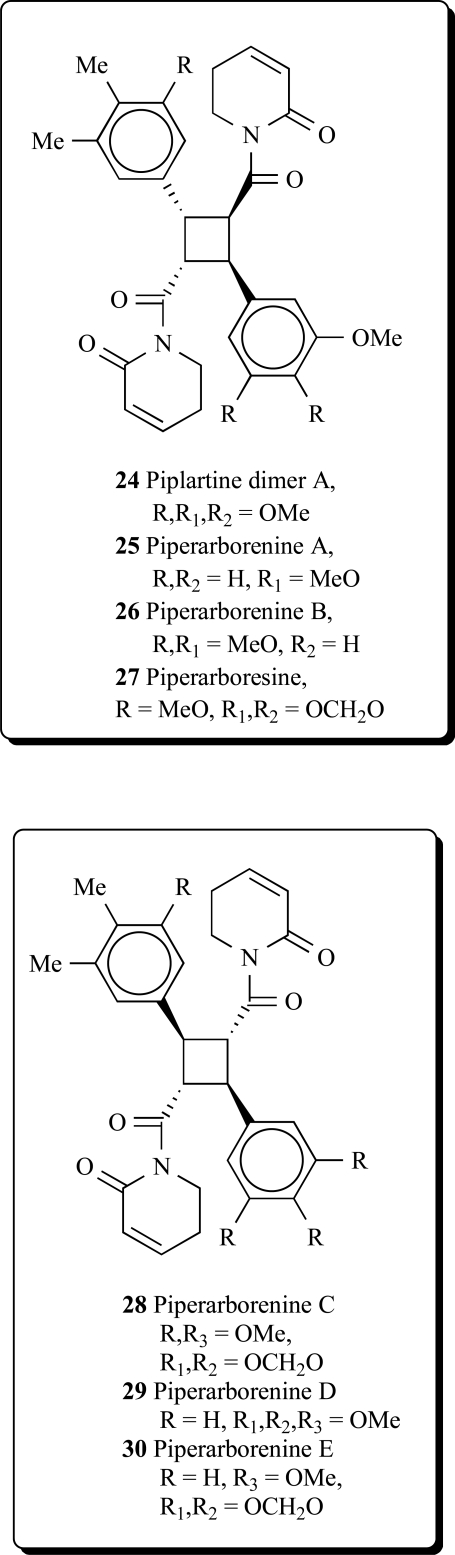


Two new pyrrolidinamide dimers have been isolated from the leaves and stem of *Piper peepuloides* and their structures established by spectral analysis as cyclobutane-2-(1,3-benzodioxol-5-methoxy-6-yl)-4-(1,3-benzodioxol-4,5-dimethoxy-6-yl)-1,3-dicarboxa-pyrrolidide (**31**) and cyclobutane-2,4-bis-(1,3-benzodioxol-5-methoxy-6-yl)-1,3-dicarboxa-pyrrolidide (**32**) [60].

Tremorgenic mycotoxins produced by fungi belonging to the genera *Penicillium*, *Aspergillus*, and *Claviceps* have potent insecticidal and growth inhibitory activity against the corn earworm (*Heliothis zea*) and the fall armyworm (*Spodoptera frugiperda*) [61-65]. Several similar indole-diterpenoid compounds with cyclobutyl unit have been isolated from these fungi species. Mycotoxins produced by 
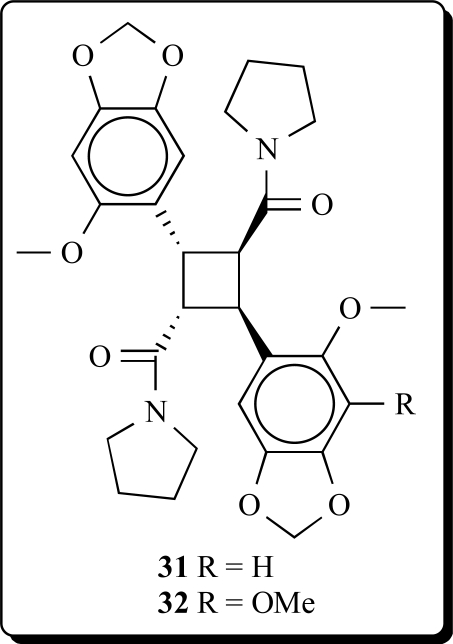
 several *Penicillium* species: *P. crustosum, P. brevicompactum, P. chrysogenum, P. expansum, P. roqueforti, P. spinulosum, P. viridicatum, P. commune, P. citrinum* and *P. solitum*, included mycophenolic acid, roquefortine C, penitrems A-F (**33-38**) and thomitrems A and E (**41** and **42**) [66-74].


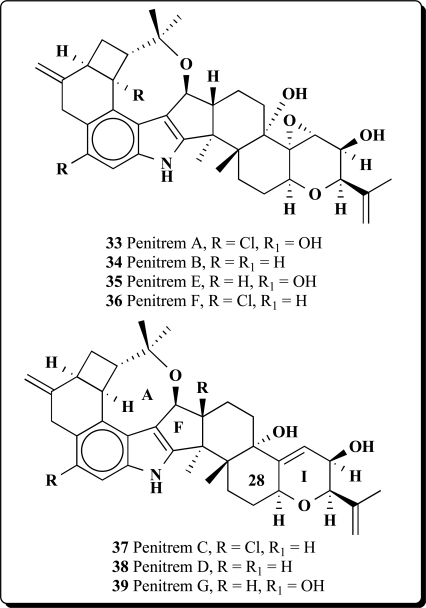


Penitrem G (**39**), a new alkaloid, has been isolated together with the already known mycotoxins penitrems A-D and F from the mycelium of *Penicillium crustosum* [75]. Penitrems A-D and F showed convulsive and insecticidal activities against both insect species. A prominent novel analog pennigritrem (**40**) of penitrem A, resolved from the tremorgenic alkaloids of a strain of *Penicillium nigricans*, showed to involve the terminal diterpenoid isoprene in a cyclization which is unique among fungal indole-diterpenoids [76].

Recently the minor metabolites, PC-M5' and PC-M6, were isolated along with the tremorgenic mycotoxins, penitrems A-F, from the mycelium of *P. crusotsum*, were found as contaminats of bread intended for school lunches in Tokyo city. Two new indoloditerpenes, PC-M4 (**43**) and PC-M5, were also isolated from the above fungus. PC-M4 has the same carbon number as the penitrems but a different cyclic ring system. PC-M5 might be a biosynthetic precursor of penitrems and PC-M4 as may also be PC-M5' and PC-M6 [77].


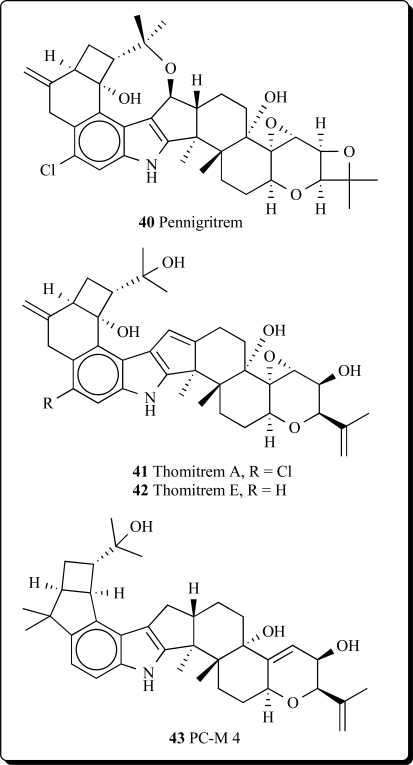


The new antiinsectan metabolite 10-oxo-11,33-dihydropenitrem B (**44**) was isolated from the sclerotia of *Aspergillus sulphureus*. Isolated alkaloid is related to the penitrems, a known group of tremorgenic fungal metabolites. A known aflavinine analog (10,23-dihydro-24,25-dehydroaflavinine) was also isolated from *A. sulphurerus sclerotia*. This is the first report of any aflavinine analog from a member of the *Aspergillus ochraceus* taxonomic group [78].

Extract from the sclerotia of *Aspergillus sulphureus* yielded four new antiinsectan compounds of the paspaline/penitrem class, and included secopenitrem B (**45**). Isolated metabolites were structurally related to penitrems. The three indole metabolites, including (**45**), exhibited potent activity against the lepidopteran crop pest *Helicoverpa zea* [79].


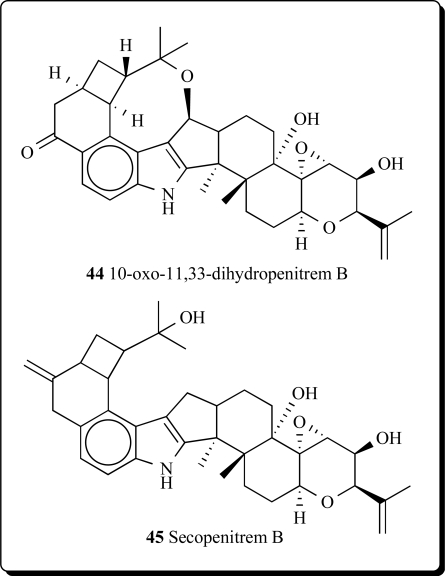


A new group of microbial metabolites, designated penitremones A-C, have been characterized by MS and NMR spectroscopy as 10-keto, and 11,33-dihydro-variants of the penitrem indole-isoprenoid skeleton. The principal metabolite penitremone A (**46**), and minor (**47**) produced with penitrem A by a *Penicillium* sp., is an isomer of penitrem E and was also similarly tremorgenic [80]. Different biological activities for penitrem A (**33**) have been studied and reported in some articles [81-88].


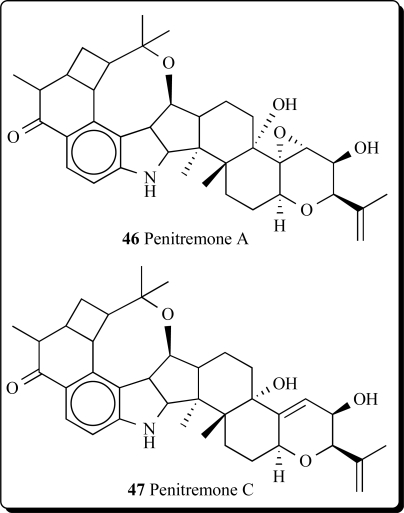


**Scheme 3:**
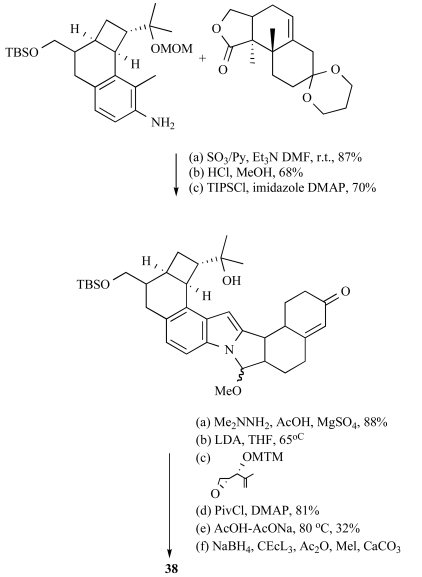


Recently, Smith III and co-workers [89] demonstrated the viability of a highly stereoselective tandem Mannich cyclization-grammine fragmentation/addition cascade, critical for assembly of the A and F rings of penitrem D (Scheme **3**). They also explored simultaneous execution of this tactic with concurrent construction of ring I. Reinvestigation of a model system provided an explanation for the unanticipated stereochemical outcome at C(28).

## PREDICTED ACTIVITIES OF ALKALOIDS ISOLATED FROM TERRESTRIAL SPECIES

Probable additional biological activities of cyclobutane-containing alkaloids were evaluated by computer prediction. For this purpose we used computer program ‘PASS’ [90-92], which predicts about 2,500 pharmacological effects, mechanisms of action, mutagenicity, carcinogenicity, teratogenicity and embryotoxicity on the basis of structural formulae of compounds. PASS predictions are based on structure-activity relationships (SAR) analysis of the training set consisting of about 60,000 drugs, drug-candidates and lead compounds. Algorithm of PASS predictions is described in detail in several publications [91,92]. Using MOL or SD files as an input for the PASS program, user may get a list of probable biological activities for any drug-like molecule as was also published recently [90].

For each activity, P_a_ and P_i_ values are calculated, which can be interpreted either as the probabilities of a molecule belonging to the classes of active and inactive compounds, respectively, or as the probabilities of the first and second kind of errors in prediction. First kind error of prediction reflects the “false-positives”, when an inactive compound is predicted to be active; and second kind error of prediction: reflects the “false-negatives”, when an active compound is predicted to be inactive.

Interpretation of the predicted results and selection of the most prospective compounds are based on flexible criteria, which depend on the purpose of particular investigation. If the user chooses a rather high value of P_a_ as a threshold for selection of probable activities, the chance to confirm the predicted activities by the experiment is high too, but many existing activities will be lost. Typically, there are several dozen biological activities in the predicted biological activity spectra; activity that is predicted with the highest probability is called “focal”. Focal biological activities for cyclobutane-containing alkaloids isolated from terrestrial sources are shown below in the Table **1**. Additional predicted biological activities for cyclobutane-containing tremorgenic mycotoxins are shown in Table **2**.

## CYCLOBUTANE-CONTAINING ALKALOIDS FROM MARINE SPECIES

Cyclobutane-containing alkaloids have also been isolated and their structures elucidated from marine algae and invertebrates, mainly from marine sponges. Some of them showed different biological activities. The water-soluble cyclobutadithymine (**48**) was extracted from the marine red alga *Porphyra yezoensis*, and showed the protective properties against UV-A irradiation [93].

John Faulkner from the University of California (Berkeley) was the first scientist who discovered an antimicrobial agent, sceptrin (**49**), from the sponge, *Agelas sceptrum*, at 1981. This alkaloid exhibited antimicrobial activity against *Staphylococcus aureus, Bacillus subtilis, Candida albicans, Pseudomonas aeruginosa*, *Alternaria* (fungus), and *Cladosporium cucumerinum*. The compound (**49**) was not toxic at 50 mg/kg [94,95]. The influence of environmental factor, namely depth and sponge-coral interactions as well as the effects of infliction of standardized damages on the production of bromopyrrolic alkaloids isolated from the sponges *Agelas dispar* and *Agelas conifera* were analyzed and sceptrin (**49**) was detected in both sponge species [96]. More recently, sceptrin and eight dimeric bromopyrrole alkaloids, nagelamides A-H, and a monomeric one, 9,10-dihydrokeramadine, have been isolated from the Okinawan marine sponge *Agelas* sp. Nagelamides A-H exhibited antibacterial activity against Gram-positive bacteria. Nagelamide G inhibited protein phosphatase 2A activity [97]. Sceptrin, ageliferine and xestospongine B, three alkaloids isolated from *Xestospongia* sp. and *Agelas novaecaledoniae* were reported as somatostatin and VIP inhibitors. Sceptrin and ageliferine showed an affinity for VIP (19.8 μM and 19.2 μM, respectively). Due to the interaction between non-peptidic compounds and somatostatin/VIP receptors, these three alkaloids could be promising agents in the research on natural non-peptidic compounds for therapeutical interventions [98].


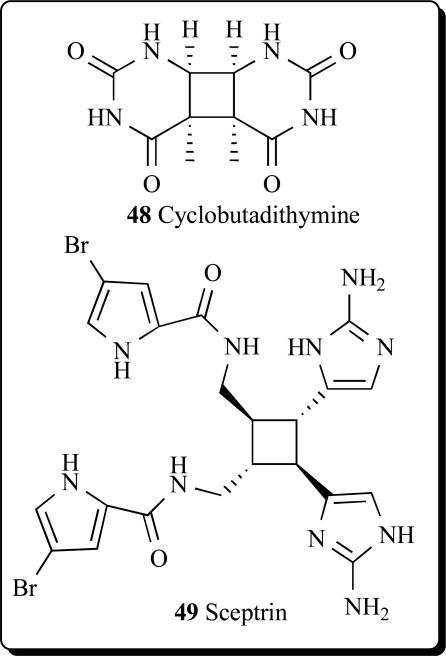


The mechanism of action of sceptrin was investigated [99]. Sceptrin has been reported to exhibit antibacterial and antifungal activities. Sceptrin demonstrated a bacteriostatic rather than bactericidal effect at the MIC on exponentially growing *Escherichia coli*. Under these conditions, the culture produced chains of cells, and incorporation of radiolabeled precursors into DNA, protein, and cell wall was unaffected, whereas incorporation of ^3^H-uridine into RNA was slightly inhibited. At concentrations higher than the MIC, sceptrin was bactericidal, inhibited the incorporation of all radiolabeled precursors, and induced the formation of unusual spheroplasts. Peptidoglycan turnover in *E. coli* appeared to be stimulated by sceptrin, as demonstrated by a release of diaminopimelic acid-containing high mol wt material. Subsequent studies of the release of K^+^ from *E. coli* and the lysis of red blood cells suggested that sceptrin disrupts the cell membranes of both prokaryotic and eukaryotic cells. Spheroplast formation may reflect a cell wall effect that occurs subsequent to membrane damage [99]. In a search for potential target sites for C_11_N_5_ compounds obtained from marine sponges of the genus *Agelas*, the authors evaluated their interaction with muscarinic acetylcholine receptors from rat brain membranes. In competition experiments with 3H-QNB, these compounds displayed the following rank order of potency: sceptrin (**49**) > oroidin, ≥ dibromosceptrin ≥ clathrodin. Sceptrin (**49**) (50 μM) was shown to be a competitive inhibitor of 3H-QNB binding as revealed Scatchard analysis. The results demonstrate the ability of these compounds to interact with multiple molecule targets in the micromolar range [100].

**Scheme 4:**
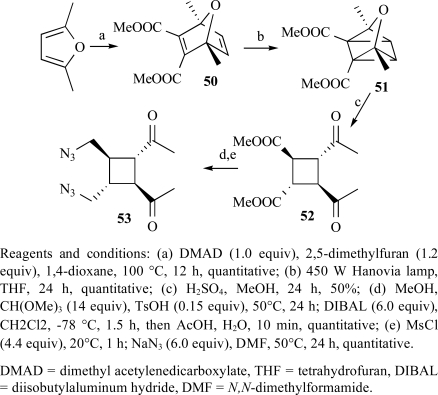


The first enantioselective total synthesis of sceptrin was reported by programming the fragmentation of an oxaquadricyclane [101]. Oxabicycle (**50**) could be obtained in quantitative yield by Alder reaction. In the [2+2] cyclization to form oxaquadricyclane (**51**), it was found that switching the solvent from diethyl ether to THF not only accelerated the reaction but also avoided potential complications due to the low boiling point of ether (Scheme **4**). The highly unstable (**51**) was taken on in crude form to the fragmentation step. The known procedure for the fragmentation of oxaquadricylane (**51**) gave only low yield of cyclobutane (**52**). As this was clearly not a viable option to support extensive synthetic efforts, a more expedient procedure was needed. It was found that the addition of diethyl ether after evaporation of methanol would cause the precipitation of cyclobutane (**52**) in ca. 50% yield, allowing expedient access to multigram quantities of (**52**). With the all-*trans *cyclobutane framework in place, (**53**) can be obtained.

**Scheme 5:**
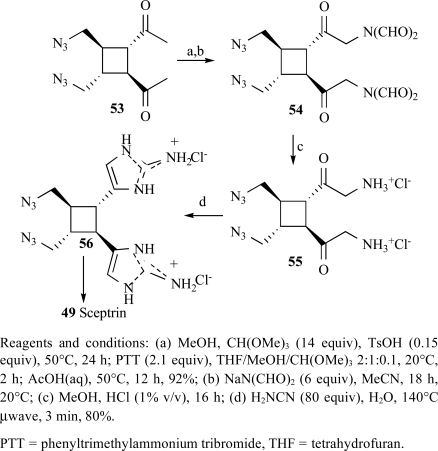


Ketalization of (**56**) under standard conditions (Scheme **5**), followed by bromination of the crude ketal with phenyltrimethyl ammonium tribromide, gave bromoketone, displacement of which gave with sodium diformamide compound (**54**), which was hydrolyzed with aqueous HCl to give (**55**) and reacted with cyanamide to form (**56**) in 80% yield over three steps, thereby completing installation of the 2-aminoimidazole, along with 5-10% of an oxazole byproduct [101].

Biologically active extracts of the Caribbean sponge *Agelas conifera* had yielded, in exhaustive studies, the diacetate salts of seven new bromopyrroles (**57-61**), as well as that of the known debromooroidin dimer sceptrin. These compounds were found to be antiviral and antibacterial and were active in barnacle settlement and biochemical prophage induction assays. The oxysceptrins are characterized by their aminoimidazolinone group, the ageliferins, by a unique cyclohexene-based skeleton [102]. Debromosceptrin (**57**), and two known pyrrole analogs (**58** and **59**) were also found in extract of the Caribbean sponge *Agelas conifera* collected from Belize [103]. Oxysceptrin (**61**) from the Okinawan marine sponge *Agelas* cf. *nemoechinata* showed active properties against actomyosin ATP-ase [104].

Nakamuric acid (**62**) and sceptrin were identified from Australian sponges belonging to the genus *Agelas* [105]. Two dimeric bromopyrrole alkaloids, nakamuric acid (**62**) and its corresponding Me ester (**63**), were isolated from the Indopacific sponge *Agelas nakamurai* along with the known 
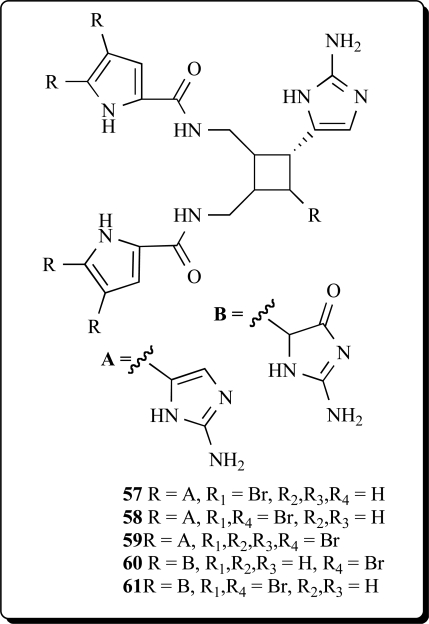
 metabolites sceptrin, debromosceptrin, and ageliferin. All compounds inhibited the growth of several Gram-positive and Gram-negative bacteria in the agar plate diffusion assay [106]. Six dimeric bromopyrrole alkaloids were isolated from a Florida Keys specimen of *Agelas conifera*. One of the constituents was identified as a new bromopyrrole metabolite, bromosceptrin (**64**) [107].


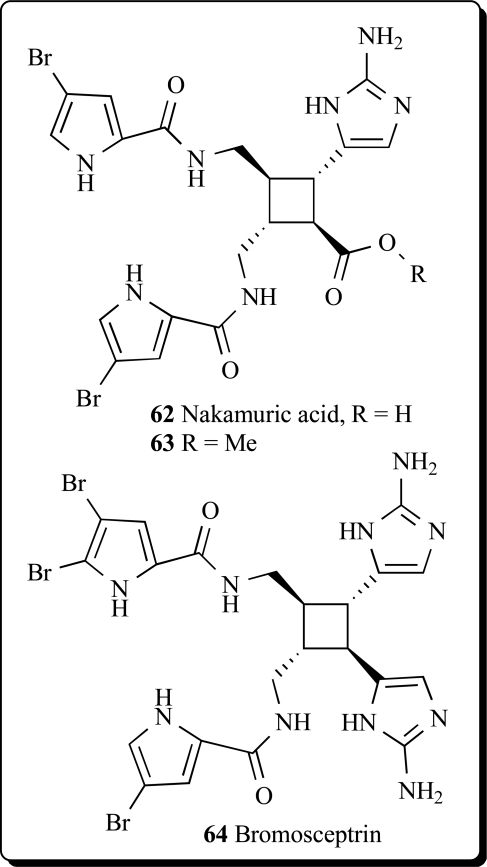


## PREDICTED ACTIVITIES FOR MARINE ALKALOIDS

Additional predicted activities for natural cyclobutane-containing alkaloids are shown in Table **3**.

## CONCLUDING REMARKS

Cyclobutane-containing alkaloids are rare group of natural products. They are mainly synthesized by different plant species, and also were detected in some marine species. A little information is known about biological activities of these metabolites. Nevertheless, reported activities for isolated compounds revealed strong antibacterial, antimicrobial, antifeedant, antinociceptive, insecticidal and others activities. The widest spectra of pharmacological activities are exhibited by isolated alkaloids, and/or their N-oxides. Using the program PASS we showed that many reported activities for cyclobutane-containing alkaloids have been predicted, including some additional biological activities.

## Figures and Tables

**Table 1:** Predicted Biological Activities for Compounds Isolated from Terrestrial Sources

No.	Drug-Likeness	Focal Activity Prediction
**1**	0.971	P_a_ = 0.964 P_i_ = 0.003 Aryl-acylamidase inhibitor
**2**	0.991	P_a_ = 0.895 P_i_ = 0.006 Dopamine release stimulant
**3**	0.965	P_a_ = 0.938 P_i_ = 0.005 Phosphatase inhibitor
**4**	0.992	P_a_ = 0.939 P_i_ = 0.005 Phosphatase inhibitor
**5**	0.958	P_a_ = 0.840 P_i_ = 0.008 Membrane integrity antagonist
**6**	0.055	P_a_ = 0.931 P_i_ = 0.001 D-amino-acid dehydrogenase inhibitor
**7**	0.832	P_a_ = 0.824 P_i_ = 0.007 Amyotrophic lateral sclerosis treatment
**8**	0.992	P_a_ = 0.728 P_i_ = 0.015 Dependence treatment
**9**	0.983	P_a_ = 0.769 P_i_ = 0.011 Dependence treatment
**10**	0.972	P_a_ = 0.820 P_i_ = 0.007 Dependence treatment
**11**	0.913	P_a_ = 0.775 P_i_ = 0.022 Neuroprotector
**12**	0.966	P_a_ = 0.855 P_i_ = 0.008 Pulmonary hypertension treatment
**13**	0.967	P_a_ = 0.848 P_i_ = 0.008 Pulmonary hypertension treatment
**14**	0.968	P_a_ = 0.841 P_i_ = 0.009 Pulmonary hypertension treatment
**15**	0.984	P_a_ = 0.794 P_i_ = 0.015 Pulmonary hypertension treatment
**16**	0.964	P_a_ = 0.838 P_i_ = 0.010 Dopamine release stimulant
**17**	0.967	P_a_ = 0.808 P_i_ = 0.015 Dopamine release stimulant
**18**	0.903	P_a_ = 0.937 P_i_ = 0.002 Cytochrome P450 inhibitor
**19**	0.889	P_a_ = 0.974 P_i_ = 0.001 Cytochrome P450 inhibitor
**20**	0.903	P_a_ = 0.940 P_i_ = 0.002 Cytochrome P450 inhibitor
**21**	0.931	P_a_ = 0.960 P_i_ = 0.002 Cytochrome P450 inhibitor
**22**	0.954	P_a_ = 0.915 P_i_ = 0.003 Cytochrome P450 inhibitor
**23**	0.956	P_a_ = 0.966 P_i_ = 0.002 Cytochrome P450 inhibitor
**24**	0.907	P_a_ = 0.919 P_i_ = 0.018 (-)-(4S)-limonene synthase inhibitor
**25**	0.882	P_a_ = 0.941 P_i_ = 0.020 (-)-(4S)-limonene synthase inhibitor
**26**	0.896	P_a_ = 0.908 P_i_ = 0.022 (-)-(4S)-limonene synthase inhibitor
**27**	0.961	P_a_ = 0.853 P_i_ = 0.008 Pulmonary hypertension treatment
**28**	0.961	P_a_ = 0.853 P_i_ = 0.008 Pulmonary hypertension treatment
**29**	0.896	P_a_ = 0.908 P_i_ = 0.022 (-)-(4S)-limonene synthase inhibitor
**30**	0.950	P_a_ = 0.866 P_i_ = 0.038 (-)-(4S)-limonene synthase inhibitor
**31**	0.875	P_a_ = 0.915 P_i_ = 0.008 Neuroprotector
**32**	0.935	P_a_ = 0.933 P_i_ = 0.007 Membrane integrity agonist

**Table 2:** Predicted Biological Activities for Tremorgenic Mycotoxins

No.	Drug-Likeness	Focal Activity Prediction
**33**	0.991	P_a_ = 0.736 P_i_ = 0.015 GABA A receptor antagonist
**34**	0.993	P_a_ = 0.740 P_i_ = 0.014 GABA A receptor antagonist
**35**	0.992	P_a_ = 0.751 P_i_ = 0.012 GABA A receptor antagonist
**36**	0.992	P_a_ = 0.724 P_i_ = 0.017 GABA A receptor antagonist
**37**	0.993	P_a_ = 0.772 P_i_ = 0.056 Phosphatase inhibitor
**38**	0.994	P_a_ = 0.765 P_i_ = 0.059 Phosphatase inhibitor
**39**	0.994	P_a_ = 0.760 P_i_ = 0.061 Phosphatase inhibitor
**40**	0.988	P_a_ = 0.810 P_i_ = 0.038 Phosphatase inhibitor
**41**	0.992	P_a_ = 0.761 P_i_ = 0.061 Phosphatase inhibitor
**42**	0.993	P_a_ = 0.734 P_i_ = 0.015 GABA A receptor antagonist
**43**	0.993	P_a_ = 0.716 P_i_ = 0.025 Ecdysone 20-monooxygenase inhibitor
**44**	0.991	P_a_ = 0.739 P_i_ = 0.014 GABA A receptor antagonist
**45**	0.993	P_a_ = 0.742 P_i_ = 0.014 GABA A receptor antagonist
**46**	0.993	P_a_ = 0.722 P_i_ = 0.018 GABA A receptor antagonist
**47**	0.993	P_a_ = 0.735 P_i_ = 0.073 Phosphatase inhibitor

**Table 3:** Predicted Biological Activities for Compounds Isolated from Marine Sources

No.	Drug-Likeness	Focal Activity Prediction
**48**	0.704	P_a_ = 0.916, P_i_ = 0.005 Antiepileptic
**49**	0.965	P_a_ = 0.669 P_i_ = 0.009 Prostaglandin E1 antagonist
**57**	0.899	P_a_ = 0.682 P_i_ = 0.008 Prostaglandin E1 antagonist
**58**	0.914	P_a_ = 0.719 P_i_ = 0.007 Prostaglandin E1 antagonist
**59**	0.921	P_a_ = 0.719 P_i_ = 0.007 Prostaglandin E1 antagonist
**60**	0.896	P_a_ = 0.616 P_i_ = 0.013 Prostaglandin E1 antagonist
**61**	0.908	P_a_ = 0.625 P_i_ = 0.010 Prostaglandin E1 antagonist
**62**	0.880	P_a_ = 0.672 P_i_ = 0.009 Prostaglandin E1 antagonist
**63**	0.837	P_a_ = 0.631 P_i_ = 0.012 Prostaglandin E1 antagonist
**64**	0.927	P_a_ = 0.682 P_i_ = 0.008 Prostaglandin E1 antagonist
